# Authorization of midwives to perform basic emergency obstetric and newborn care signal functions in Argentina, Ghana, and India: A multi-country validation study of a key global maternal and newborn health indicator

**DOI:** 10.1371/journal.pone.0283029

**Published:** 2023-04-20

**Authors:** Sowmya Ramesh, Suchandrima Chakraborty, Richard M. Adanu, Delia A. B. Bandoh, Mabel Berrueta, Jewel Gausman, Nizamuddin Khan, Ernest Kenu, Ana Langer, Carolina Nigri, Magdalene A. Odikro, Verónica Pingray, Niranjan Saggurti, Paula Vázquez, Caitlin R. Williams, R. Rima Jolivet

**Affiliations:** 1 Population Council, New Delhi, India; 2 Department of Population, Family, and Reproductive Health, University of Ghana School of Public Health, Accra, Ghana; 3 Department of Epidemiology and Disease Control, University of Ghana School of Public Health, Accra, Greater Accra, Ghana; 4 Institute for Clinical Effectiveness and Health Policy, Buenos Aires, Argentina; 5 Women and Health Initiative, Department of Global Health and Population, Harvard University T.H. Chan School of Public Health, Boston, Massachusetts, United States of America; 6 Department of Health Science, Kinesiology, and Rehabilitation, Universidad Nacional de La Matanza, Buenos Aires, Argentina; 7 Department of Maternal & Child Health, Gillings School of Global Public Health, University of North Carolina at Chapel Hill, Chapel Hill, North Carolina, United States of America; Johns Hopkins University Bloomberg School of Public Health, UNITED STATES

## Abstract

**Background:**

Midwives’ authorization to deliver the seven basic emergency obstetric and newborn care (BEmONC) functions is a core policy indicator in global monitoring frameworks, yet little evidence supports whether such data are captured accurately, or whether authorization demonstrates convergence with midwives’ skills and actual provision of services. In this study, we aimed to validate the data reported in global monitoring frameworks (criterion validity) and to determine whether a measure of authorization is a valid indicator for BEmONC availability (construct validity).

**Methods:**

We conducted a validation study in Argentina, Ghana, and India. To assess accuracy of the reported data on midwives’ authorization to provide BEmONC services, we reviewed national regulatory documents and compared with reported country-specific data in Countdown to 2030 and the World Health Organization Maternal, Newborn, Child and Adolescent Health Policy Survey. To assess whether authorization demonstrates convergent validity with midwives’ skills, training, and performance of BEmONC signal functions, we surveyed 1257 midwives/midwifery professionals and assessed variance.

**Results:**

We detected discrepancies between data reported in the global monitoring frameworks and the national regulatory framework in all three countries. We found wide variations between midwives’ authorization to perform signal functions and their self-reported skills and actual performance within the past 90 days. The percentage of midwives who reported performing all signal functions for which they were authorized per country-specific regulations was 17% in Argentina, 23% in Ghana, and 31% in India. Additionally, midwives in all three countries reported performing some signal functions that the national regulations did not authorize.

**Conclusion:**

Our findings suggest limitations in criterion and construct validity for this indicator in Argentina, Ghana, and India. Some signal functions such as assisted vaginal delivery may be obsolete based on current practice patterns. Findings suggest the need to re-examine the emergency interventions that should be included as BEmONC signal functions.

## Introduction

Obstetric complications during pregnancy, childbirth, and/or the postnatal period can lead to maternal mortality without timely intervention. An estimated 295,000 women died worldwide during pregnancy, childbirth, or the first 42 days following pregnancy in 2017, with the majority of deaths occurring in low- and lower middle-income countries [1].

Reducing maternal mortality is a key global priority. The United Nations (UN) Sustainable Development Goals (SDG) Target 3.1 establishes a global average maternal mortality ratio target of <70 deaths per 100,000 live births by 2030 [2]. Undermining this goal, many places still lack universal access to high-quality healthcare services [3]. Indeed, skilled care is crucial for saving lives and promoting health of women and newborns [4]. Evidence suggests that >90% of births in high- and upper middle-income countries occur in the presence of a skilled birth attendant, while <50% of all births in many low- and lower middle-income countries are assisted by skilled health personnel [5]. Shortage of skilled health workers and the lack of an enabling environment in which they can practice (including essential equipment and commodities but also a supportive regulatory environment and a respectful workplace) limit access to quality maternal and newborn healthcare [6–8].

The increased pressure on health systems in low- and middle-income countries stokes a growing recognition of the critical role of midwives in ensuring skilled birth attendance [4, 9]. The Lancet series on midwifery states that 83% of all maternal deaths, stillbirths, and newborn deaths can be averted with midwifery care, thereby highlighting the benefits of introducing and integrating midwives into health systems [10, 11]. Midwives trained to global standards can deliver 87% of essential maternal and newborn health care [12]. However, to implement these services safely and effectively, midwives need regulatory support and recognition from health systems, including authorization to perform essential tasks. Regulatory mechanisms protect public safety and promulgate standards of care that enable the public to trust midwifery professional practice. In many countries, midwives are not authorized to perform tasks that are part of the midwifery scope of practice as per global standards [10, 11].

To address the most significant direct causes of maternal and neonatal mortality, the World Health Organization (WHO), United Nations Children’s Fund, and United Nations Population Fund introduced basic emergency obstetric and neonatal care (BEmONC) signal functions in 1997 [13, 14]. BEmONC signal functions are a package of seven essential medical and surgical lifesaving clinical interventions (**Box 1**). Numerous quasi-experimental or experimental studies and several systematic reviews indicate that these essential signal functions are cost-effective priority interventions to reduce maternal and neonatal morbidity and mortality [15–18]. Because midwives are the cadre of frontline health workers best suited to deliver the majority of maternal and newborn health interventions [12, 19] including all BEmONC signal functions [10, 11], ensuring that midwives in all settings have the necessary support through education, regulation, and professional recognition to provide all BEmONC interventions presents an important opportunity to improve maternal/neonatal outcomes.

**Box 1. BEmONC signal functions**

1. Administer parenteral antibiotics
2. Administer uterotonic drugs
3. Administer parenteral anticonvulsants
4. Manual removal of the placenta
5. Removal of retained products of conception
6. Assisted vaginal delivery
7. Neonatal resuscitations with bag and mask

The Ending Preventable Maternal Mortality (EPMM) Strategies monitoring framework—a direction-setting report released by the WHO for reducing maternal mortality in the era of SDGs—prioritized the core indicator “*Midwives are authorized to deliver basic emergency obstetric and newborn care*” [20]. This indicator was prioritized for its potential to advance country leadership for ending preventable maternal deaths through supportive legal and regulatory mechanisms, an EPMM Key Theme [21]. The intended use of the indicator is to monitor the enabling regulatory environment that supports provision of all basic emergency services by midwives and midwifery professionals. Thus, it is important that the indicator provides a robust measure of both the policy and the practice it is intended to support to inform advancement in each of these areas.

Midwives’ authorization to perform BEmONC signal functions is a tracer indicator for skilled care coverage of the most essential emergency interventions to save lives, serving as a proxy for “know-can-do”. This model demonstrates the relationship between provider knowledge, enabling factors, and intervention performance in healthcare and has been used to diagnose and address low quality of care [22, 23]. The BEmONC indicator specifically monitors the “can” part of the phrase. The assumption is that midwives’ authorization to perform signal functions, i.e., legal authorization at national level (“can”) will converge with their acquisition of the knowledge to perform the functions, i.e., required education and training (“know”), and this will result in actual performance (“do"). Yet, policy-level indicators are rarely systematically validated through research to ensure they achieve their intended measurement goals [24, 25]. The aim of this study is to assess the validity of a key measure of midwifery workforce ability to provide essential care. The overall research goal is to provide data that can strengthen the validity of this indicator and furnish information to guide improvements to both policy and practice frameworks.

## Materials and methods

### Study design

This validation study used various approaches to data collection, including secondary data compilation through a policy review, and primary data collection through a cross-sectional survey of midwives and midwifery professionals. Using policy and individual data, respectively, we aimed to address two validation questions:

1. Does the national regulatory framework (laws, guidelines, policies) in countries that authorize midwives and midwifery professionals to deliver emergency maternal and newborn care match what has been reported to Countdown to 2030 (hereafter, Countdown) and the WHO Maternal, Newborn, Child, and Adolescent Health (MNCAH) Policy Survey on all seven BEmONC signal functions? [26].
2. Do midwives and midwifery professionals report actual performance of tasks in the last 90 days for signal functions that they are authorized to perform?

### Study setting

The study was conducted in Argentina, Ghana, and India, representing diverse geographic settings. The research countries were purposively selected as part of the parent research project that includes this study to represent low- and middle-income countries (LMICs) from a mix of regions (Asia, Africa, and the Latin America/Caribbean) with significant national burden of maternal mortality. All three countries have different health systems and variations in health system performance both nationally and between states/regions. In each country, primary data collection was from four selected districts/provinces based on a composite index of key maternal health indicators reflecting variations in health system performance. Based on this index, we first selected one state/region in the highest performing and lowest performing quartiles. Within each state/region, we then selected one district/province each in the highest performing and lowest performing quartiles (terciles in Argentina due to low population density). Details of the sampling plan and selection of districts for this study are described elsewhere [27].

Thereafter, a multi-stage standardized sampling plan was used to select facilities from which to recruit participants for primary data collection. In each selected district/province, we obtained a list of all public health facilities and private facilities registered with the government that provide the maternal health-related services enumerated in the WHO MNCAH Policy Survey from the district health department. Thereafter, inclusion of facilities differed to some extent across countries. In Ghana, all primary and secondary level health facilities that provided birth-care and/or other maternal health-related services as per the WHO MNCAH Policy Survey were selected.

In India, all secondary- and tertiary-level facilities were included, as well as a random sample of 20 primary health care facilities that were designated as birthing facilities were selected given the large number of facilities at this level. We performed a sample size calculation to determine our sample size of 20 facilities. Given that the 20 facilities were randomly selected, we do not anticipate our estimates being subject to bias.

In Argentina, facilities that offered all categories of maternal health-related services enumerated in the WHO MNCAH Policy Survey and that employed midwives were included. From this list, for feasibility reasons given large distances in the provinces, a purposive sample of facilities at all three levels of health system (primary, secondary, and tertiary) was selected.

### Data collection

To address the first validation question (criterion validity), we performed a cross-sectional review of primary national policies, laws, and regulations, which we considered to be the reference or “gold” standard for verification of what was reported to global monitoring frameworks, as described by Benova et al. (2020) [28]. We systematically searched for national policies, laws, and regulations through a comprehensive desk review of relevant source documents in Argentina, Ghana, and India. In Argentina, we searched the website of "Sistema Argentino de Información Jurídica" of the Ministry of Justice and Human Rights for all laws related to midwifery care and scope of practice. In Ghana, we searched the websites of the Ghana Health Service, Nurses and Midwifery Council, and the Ministry of Health using key words related to midwives and their scope of practice for relevant documents. In India, we searched the websites of the Ministry of Health and Family Welfare and the Indian Nursing Council using key words related to midwifery and associate midwifery professionals and their scope of practice. We further manually searched relevant paper Nursing Council documents. We also consulted subject experts to ensure that all relevant documents were collected and reviewed, considering all policies and guidelines that were applicable during the period from 2017–2020.

Secondary data collection in all three countries took place between May and September 2020. Data analysis took place from October to November 2020. We extracted and analyzed country-specific data from the Countdown to 2030 country profiles from the 2017 report, and responses to the 2018 WHO MNCAH Policy Survey during the data collection period. We collected and reviewed source documents, considering all policies and guidelines that were applicable during the period from 2017–2020 [26].

To standardize the review process across countries, we developed a data extraction form with fields for each signal function. Responses for each signal function were coded as authorized (if mentioned in the source document as “authorized”), not authorized (if mentioned in the source document as “not authorized”), or not specified (if not mentioned in the source document) in the documents reviewed. Two team members in each country reviewed the documents and coded responses independently in the data extraction form. Discrepancies were resolved through re-review of documents and consensus. As needed, a third member of the team resolved disagreements based on review of documents.

To address the second validation question (construct validity), we performed a task survey of midwife/midwifery professionals in study district/province facilities. Survey questions were based on the seven signal functions and were translated to the local language of each country. We conducted cognitive testing of the instruments to evaluate whether respondents understood the questions and could accurately respond. We incorporated learnings from the cognitive study into the questionnaire.

### Study participants

A census of all midwifery professionals who met our study definition was recruited from all eligible facilities, as described above. For feasibility reasons, we made a provision that in facilities where more than 50 health workers were eligible, we would recruit a random sample of 50 participants. In Ghana, only one facility had 60 midwives employed. In India, two facilities had more than 50 midwives (one with 59 and one with 67). In Argentina, no facilities had more than 50 midwives.

The staffing coordinator in each selected facility provided a list of all currently employed healthcare workers who planned, managed, provided, and evaluated (or just provided) midwifery care services, basic health care, and advice during and after pregnancy and childbirth, including family planning and sexual and reproductive health services, as per the International Labour Organization (ILO) classification system for midwifery professionals and associate professionals. Healthcare workers were eligible to participate in the survey if they met the criteria of the ILO International Standard Classification of Occupations (ISCO-8) for midwifery professionals and/or associate professionals, regardless of their credentials or job title [29]. We screened all healthcare workers from all selected facilities in each country for eligibility–in person in Ghana, and by telephone in Argentina and India—to verify whether they met the ILO standard classification for midwifery professionals or midwifery associate professionals based on their job functions. We recruited a census of all eligible participants from facilities that employed <50 midwifery professionals. In facilities with >50 eligible employees, we recruited a random sample of 50 participants. In total, 77 respondents completed the interview in Argentina, 414 in Ghana, and 766 in India. Given the census-based sample, and where a census was not possible, large and randomly selected sample, we did not perform a sample size calculation and do not anticipate our results being subject to bias.

Data were collected from midwifery professionals in July 2020–August 2021. We used different data collection methods in the three countries based on the COVID-19 situation. In Ghana, we conducted in-person interviews; in India, interviews were conducted via telephone by trained research investigators in the local language of the district using a structured questionnaire. In Argentina, the same questionnaire was self-administered through electronic surveys. The survey included questions to collect participant socio-demographic characteristics. Participants were asked to report whether they believe they possess the skills necessary to perform BEmONC signal functions. Additional questions focused on the frequency and recency of behaviors related to each signal function and reasons for non-performance of these behaviors in their current job.

### Ethical considerations

The study was approved by the ethical review board in the Office of Human Research Administration at Harvard University (IRB19-1086). Each country also obtained approval from local institutional ethical review boards. For Argentina, local institutional review boards approved the study (Comité de Ética de la Investigación de la Provincia de Jujuy–Approval ID Not applicable. Comisión Provincial de Investigaciones Biomédicas de la Provincia de Salta–Approval ID 321-284616/2019. Consejo Provincial de Bioética de la Provincia de La Pampa–Approval ID Not applicable. Comité de Ética Central de la Provincia de Buenos Aires–Approval ID 2919-2056-2019). In India, the local institutional review board Sigma-IRB (IRB Number: 10052/IRB/19-20) approved the study and the Ghana Health Service Ethics Review Committee (GHS-ERC022/08/19) approved the study in Ghana.

All participants were informed about the study objectives and their right to refuse to participate in the study or withdraw anytime during the interview. We encouraged and answered participants’ questions before initiating interviews. Written informed consent was obtained from all participants before data collection. We enacted robust procedures to ensure confidentiality, voluntary participation, and adequate data protection. Participants were recruited in such a manner to ensure anonymity so that their colleagues and supervisors did not know about their participation in the study.

### Measures

Background characteristics included were age, sex (male/female), education (certificate program, technical degree/diploma, university degree), duration of pre-service training (<2 years, 2 years, 3 years, 4 years, >4 years), years in service, type of facility where they were employed at the time of interview (primary, secondary, tertiary), and number of hours worked per week (>40 hours, 40 hours, <40 hours).

For BEmONC signal functions, participants were asked whether they had the skills to perform each function: (1) administration of parenteral antibiotics, (2) administration of uterotonic drugs (oxytocin, misoprostol), (3) administration of parenteral anticonvulsants, (4) manual removal of placenta, (5) removal of retained products of conception, (6) assisted vaginal delivery (vacuum extraction, forceps), and (7) neonatal resuscitation with bag and mask. Participants who reported having the skills were asked how they obtained them and about their performance of those functions in the past 90 days. In addition, we also assessed reasons for non-performance of signal functions in their current job.

### Analysis

To answer the first validation question, we compared the national policy, legal, and regulatory framework on midwifery authorization to perform BEmONC signal functions in each country with corresponding data reported in the Countdown country profile and WHO MNCAH Policy Survey. We compared each signal function using data from the study-based data extraction form, which was filled by comprehensive review of several source documents for the same political unit, with data reported in global monitoring frameworks. Variations between national policy and the two global monitoring frameworks were identified and documented.

To understand the extent to which midwives and midwifery professionals practiced signal functions for which they were authorized, we calculated the percentage of individuals who reported having the skills and performing those tasks in the past 90 days. We examined the percentage of midwives who reported possessing all skills associated with the BEmONC signal functions they were authorized to perform in each country. All the data from this analysis are presented at aggregate level as well as by facility type. Variation between midwifery professionals’ authorization, skills, and practice patterns is reported in the paper. Stata software version 16.0 was used for all statistical analysis.

## Results

### Secondary data analysis

The number of signal functions authorized by the national regulatory framework varied by country. In Argentina, national regulatory framework authorizes midwives to perform only two signal functions, whereas in Ghana, it authorizes them to perform all seven signal functions except dilatation and curettage for removal of retained products of conception and forceps delivery, while in India, the national regulatory framework authorizes midwifery professionals to perform four signal functions. We detected discrepancies between the source documents in each country and the information reported in Countdown to 2030 and the WHO MNCAH Policy Survey, as well as variations between Countdown and the WHO MNCAH Policy Survey, for all three countries (**Tables 1–3**). The national regulatory framework in Argentina authorizes midwives to perform two signal functions (2 and 4); however, information in the Countdown country profile reflected authorization for three signal functions (4, 6, and 7), and the WHO MNCAH Policy Survey indicated authorization for four functions (1, 4, 6, and 7). For Argentina, the Countdown data matched the national regulatory framework for only one signal function, and the WHO MNCAH Policy Survey data matched the national regulatory framework for three signal functions (**Table 3**). For Ghana, the national regulatory framework indicates midwives’ authorization to perform all signal functions except dilatation and curettage for removal of retained products of conception and forceps delivery; while the WHO MNCAH Policy Survey data matched data from country source documents, the Countdown country profile did not mention authorization or labeled some signal functions “not applicable”. In India, the national regulatory framework authorizes midwives/midwifery professionals to perform four signal functions (1, 2, 3, and 7); while Countdown did not document such information, and the WHO MNCAH Policy Survey data matched with national regulatory framework for only one signal function, with no additional information reported for the remaining signal functions (7).

**Table 1:** National policy authorizing midwives to perform BEmONC signal functions.

BEmONC signal function	Validation data (cadre of midwife authorized)											
	Argentina				Ghana				India			
	Midwife	Associate Midwife	Midwifery Professional (Nurse-midwife)	Associate Midwifery Personnel	Midwife	Associate Midwife	Midwifery Professional (Nurse-midwife)	Associate Midwifery Personnel	Midwife	Associate Midwife	Midwifery Professional (Nurse-midwife)	Associate Midwifery Personnel
Administer parenteral antibiotics	✘	✘	NA	NA	✓	✓	✓	✓	NA	NA	✓	✓
Administer uterotonic drugs	✓	✘	NA	NA	✓	✓	✓	✓	NA	NA	✓	✓
Oxytocin	✓	✘	NA	NA	✓	✓	✓	✓	NA	NA	✓	✓
Misoprostal	✘	✘	NA	NA	✓	✓	✓	✓	NA	NA	✓	✓
Administer parenteral anticonvulsants	✘	✘	NA	NA	✓	✓	✓	✓	NA	NA	✓	✓
Manual removal of placenta	✓	✘	NA	NA	✓	✓	✓	✓	NA	NA	✘	✘
Removal of retained products of conception	✘	✘	NA	NA	✓	✓	✓	✓	NA	NA	✘	✘
Manual	✘	✘	NA	NA	✓	✓	✓	✓	NA	NA	✘	✘
Dilation and curettage	✘	✘	NA	NA	✘	✘	✘	✘	NA	NA	✘	✘
Assisted vaginal delivery	✘	✘	NA	NA	✓	✓	✓	✓	NA	NA	NA	NA
Vacuum extraction	✘	✘	NA	NA	✓	✓	✓	✓	NA	NA	NA	NA
Forceps	✘	✘	NA	NA	✘	✘	✘	✘	NA	NA	NA	NA
Neonatal resuscitation with bag and mask	✘	✘	NA	NA	✓	✓	✓	✓	NA	NA	✓	✓
Total number of signal functions authorized	2	0	-	-	7*	7*	7*	7*	-	-	4	4

NA: “Not applicable” in the country context. ✓: Authorized by national regulatory framework. ✘: Not authorized by national regulatory framework. *Except dilatation and curettage for removal of retained products of conception and forceps delivery

**Table 2:** Reported authorization for BEmONC signal functions in Countdown to 2030 and WHO MNCAH Policy Survey, by country.

BEmONC signal function	Countdown to 2030 (midwife authorization)			WHO MNCAH Policy Survey (cadre of midwife authorized)								
	Argentina	Ghana	India	Argentina			Ghana			India		
				Nurse	Midwife	Nurse-midwife	Nurse	Midwife	Nurse-midwife	Nurse	Midwife	Nurse-midwife
Administer parenteral antibiotics				No	Yes	No	Yes	Yes	Yes			
Administer uterotonic drugs	NA	NA	NA									
Oxytocin				No	No	No	Yes	Yes	Yes			
Misoprostol	NA	NA	NA	No	No	No	Yes	Yes	Yes			
Administer parenteral anticonvulsants				No	No	No	Yes	Yes	Yes			
Manual removal of placenta	Yes			No	Yes	No	Yes	Yes	Yes			
Removal of retained products of conception				No	No	No	Yes	Yes	Yes			
Manual	NA	NA	NA	No	No	No	Yes	Yes	Yes			
Dilation and curettage	NA	NA	NA				No	No	No			
Assisted vaginal delivery	Yes			No	Yes	No	Yes	Yes	Yes			
Vacuum extraction	NA	NA	NA				Yes	Yes	Yes			
Forceps	NA	NA	NA				No	No	No			
Neonatal resuscitation with bag and mask	Yes			Yes	Yes	No	Yes	Yes	Yes	Yes	Yes	Yes

Gray color: Information is not mentioned in the database. NA: Information is mentioned as “not applicable” in the database. Yes: Specified as “yes” in the database, indicating authorized by country. No: Specified as “no” in the database, indicating not authorized by country.

**Table 3:** Match between national regulatory frameworks for BEmONC signal functions and reported authorization in Countdown to 2030 and WHO MNCAH Policy Survey, by country.

BEmONC signal function	Countdown to 2030 (midwife authorization)			WHO MNCAH Policy Survey		
	Argentina	Ghana	India	Argentina	Ghana	India
Administer parenteral antibiotics				No Match	Match	
Administer uterotonic drugs	No Match	No Match	No Match	No Match	Match	
Administer parenteral anticonvulsants				Match	Match	
Manual removal of placenta	Match			Match	Match	
Removal of retained products of conception	No Match	No Match	No Match	Match	Match	
Assisted vaginal delivery	No Match	No Match	No Match	No Match	Match	
Neonatal resuscitation with bag and mask	No Match			No Match	Match	Match

Gray color: Information is not mentioned in the global database for comparison. No Match: if there was discrepancy between the national policy and global monitoring framework. Match: if no discrepancy between national policy and global monitoring framework irrespective of whether the signal function was authorized or not authorized in the country.

### Socio-demographic characteristics of participants

**Fig 1:**
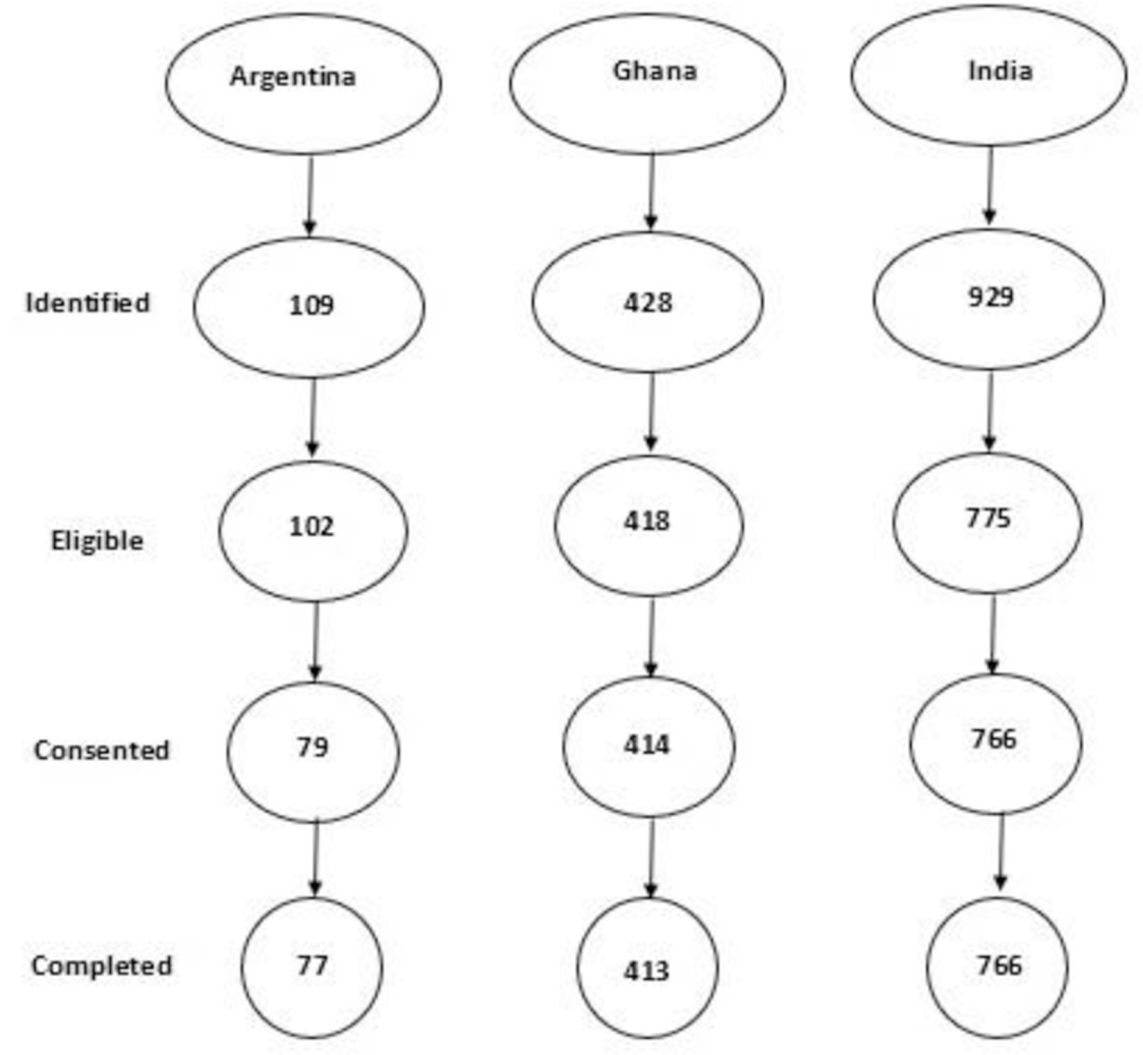
Flowchart of participant selection in Argentina, Ghana, and India.

Out of the total health workers identified by staffing coordinators, 93.6% in Argentina, 97.7% in Ghana, and 83.4% in India were eligible to participate in the survey. Of eligible health workers, 75.5% in Argentina, 98.8% in Ghana, and 98.8% in India completed the survey (**Fig 1**). More than 85% of interviewed participants were female in all three countries (**Table 4**). On average, across settings, participants were over 30 years of age, had over 7 years of experience, a majority were employed full time in their current place of work, and most reported working >40 hours per week. Significant differences between settings were observed in educational attainment and facility type for participants’ current place of employment. Nearly all participants in Argentina had a university degree (99%), while most participants in India had a technical degree or diploma (84%), and respondents in Ghana either attended a certificate program (50%) or had a technical degree or diploma (43%). Most participants in Argentina worked in tertiary care facilities (79%), whereas most participants worked in secondary care facilities in India (55%) or primary care facilities in Ghana (85%).

**Table 4:** Characteristics of participants in task survey.

**Characteristic**	**Argentina**	**Ghana**	**India**
Total number of participants (n)	77	414	766
Mean age (SD)	41.1 (10.4)	34.2 (8.9)	36.2 (9.5)
Gender % (n)			
Male	6.5 (5)	14.0 (58)	3.3 (25)
Female	93.5 (72)	86.0 (356)	96.7 (741)
Other	0.0 (0)	0.0 (0)	0.0 (0)
Refused	0.0 (0)	0.0 (0)	0.0 (0)
Credentials % (n)			
Certificate program	0.0 (0)	49.8 (206)	9.3 (71)
Technical degree or diploma	1.3 (1)	42.8 (177)	84.2 (645)
University degree	98.7 (76)	7.3 (30)	6.5 (50)
Refused	0.0 (0)	0.2 (1)	0.0 (0)
Duration of pre-service training % (n)			
<2 years	0.00 (0)	3.6 (15)	11.9 (91)
2 years	0.00 (0)	53.9 (223)	8.2 (63)
3 years	10.4 (8)	34.3 (142)	77.8 (596)
4 years	28.6 (22)	4.1 (17)	2.1 (16)
>4 years	59.7 (46)	2.2 (9)	0.0 (0)
Refused	1.3 (1)	1.9 (8)	0.0 (0)
Mean years in service (SD)	13.1 (8.4)	7.4 (7.6)	10.2 (8.7)
Facility type % (n) *some respondents work in >1 facility			
Primary care	40.2 (31)	85.3 (353)	27.2 (208)
Secondary care	35.1 (27)	14.7 (61)	55.5 (425)
Tertiary care	79.2 (61)		17.4 (133)
Employment			
Full-time	68.8 (53)	98.1 (405)	80.7 (618)
Part-time	28.6 (22)	0.7 (3)	19.3 (148)
Refused	2.6(2)	1.2 (5)	0.0 (0)
Number of hours worked per week			
>40 hours	55.9 (43)	70.1 (290)	60.8 (466)
40 hours	29.8 (23)	17.6 (73)	7.3 (56)
<40 hours	14.3 (11)	12.3 (51)	31.9 (244)

### Comparison between authorization and self-reported competency

We observed wide variation between authorization to perform signal functions documented in the source documents for each country and midwives’/midwifery professionals’ self-reported skills to perform each of them (**Table 5**). Further, we found variation in the skills reported by midwives in the same country for different authorized signal functions. For example, in Argentina, >90% of respondents reported that they had the skills to administer oxytocin. However, only 66% of participants reported having the skills to perform manual removal of the placenta, although both these signal functions are authorized by the national regulatory framework. Additionally, only 62% reported that they had all necessary skills to execute every authorized signal function. In Ghana, >75% of participants stated they had the skills to administer parenteral oxytocin, whereas <40% reported the skills to conduct assisted vaginal delivery via vacuum extraction, although both signal functions are authorized by the national regulatory framework. Further, only 17% of the respondents reported that they had all the skills to perform every authorized signal function. In India, >90% of respondents reported that they had the skills to perform the signal functions for which they are authorized. Administering parenteral oxytocin was the only signal function that was authorized across all the three countries, and it was also the most commonly reported skill by the participants in every setting.

**Table 5:** Status of national policy, skill level, and practice of BEmONC signal functions among midwives/midwifery professionals by country.

BEmONC signal function	Argentina			Ghana			India		
	National policy validation data	Skills and performance		National policy validation data	Skills and performance		National policy validation data	Skills and performance	
	National authorization to perform task	Having the necessary skills	Performed the skill in last 90 days *	National Authorization to perform task	Having the necessary skills	Performed the skill in last 90 days *	National Authorization to perform task	Having the necessary skills	Performed the skill in last 90 days *
Administer parenteral antibiotics % (n)	✘	74.0 (57)	75.4 (43)	✓	74.6 (309)	69.3 (214)	✓	96.3 (738)	86.4 (638)
Administer uterotonic drugs									
Parenteral oxytocin % (n)	✓	93.5 (72)	83.3 (60)	✓	75.4 (312)	74.4 (232)	✓	99.5 (762)	85.3 (650)
Parenteral misoprostol % (n)	✘	64.5 (49)	59.1 (29)	✓	62.8 (260)	69.6 (181)	✓	97.7 (749)	68.6 (514)
Administer parenteral anticonvulsants % (n)	✘	40.0 (30)	26.6 (8)	✓	65.2 (270)	49.3 (133)	✓	93.7 (718)	41.9 (301)
Manual removal of placenta % (n)	✓	66.2 (51)	27.5 (14)	✓	64.7 (268)	51.1 (137)	✘	28.2 (216)	37.5 (81)
Manual removal of retained products of conception % (n)	✘	50.7 (39)	51.2 (20)	✓	62.3 (258)	55.4 (143)	✘	64.1 (491)	62.9 (309)
Assisted vaginal delivery									
Vacuum extraction % (n)	✘	1.3 (1)	0.0 (0)	✓	39.9 (165)	35.8 (59)	✘	30.1 (231)	1.3 (3)
Forceps % (n)	✘	1.3 (1)	0.0 (0)	✘	25.1 (104)	31.7 (33)	✘	44.6 (342)	7.0 (24)
Neonatal resuscitation with bag and mask % (n)	✘	24.7 (19)	0.0 (0)	✓	68.8 (285)	56.8 (162)	✓	98.5 (755)	58.2 (440)
ALL signal functions for which they have authorization % (n)		62.0 (49)	16.5 (13)		16.9 (70)	22.9 (16)	** **	90.1 (690)	30.6 (211)

*: Among those who reported having the necessary skills. ✓: Authorized by national regulatory framework to perform. ✘: Not authorized by national regulatory framework to perform. % indicates percentage of midwives reporting having the skills, performing that skill.

### Self-reported competency and performance of authorized signal functions

Midwives who reported that they had the necessary skills to perform a signal function were further asked whether they had done so in the past 90 days. In all three countries, despite legal authorization to perform certain BEmONC signal functions, a large proportion of midwifery professionals reportedly did not perform them (**Table 5**). We observed wide variability in the performance of authorized signal functions in the past 90 days among midwives who reported that they had the necessary skills. In Argentina, the rate of performance ranged from 28% (for manual removal of the placenta) to 83% (for oxytocin administration). In Ghana, performance ranged from 36% (for vacuum extraction) to 75% (for oxytocin administration). In India, performance ranged from 42% (to administer parenteral anticonvulsants) to 86% (for parenteral antibiotic administration) in the past 90 days.

Only 23% of midwives in Ghana and 31% of midwives in India reported that they performed all signal functions for which they were authorized as per country-specific regulations in the past 90 days, while 17% in Argentina reported performing all authorized signal functions. Among those who reported performing all authorized signal functions, a high proportion were those with >10 years of experience. For example, 46% of those who reported performing all authorized signal functions in India had >10 years of experience. Administering parenteral oxytocin was the authorized signal function that participants most commonly reported they had performed in the past 90 days in all three settings. In India, administering parenteral antibiotics was reported equally frequently by the midwifery professionals in the recent past.

### Skills and performance of authorized signal functions by facility type

We observed variability between reported skills and performance of authorized signal functions by type of health facility (**S1 Table**). In Ghana, a high proportion of midwives posted at secondary care facilities reported both that they had the skills to perform authorized BEmONC signal functions and actually performed those functions in the past 90 days. In Argentina, while a high proportion of midwives who worked in tertiary care facilities reported having the skills to perform authorized signal functions, those working in secondary care facilities reported higher rates of actual performance in the past 90 days. In India, performance by type of facility varied for each signal function. A higher proportion of midwives working in secondary care facilities compared to midwives in other types of facilities reported administering antibiotics (90%), oxytocin (89%), and anticonvulsants (45%) in the past 90 days; however, a higher proportion of midwifery professionals working in tertiary care facilities (71%) reported providing neonatal resuscitation relative to those working in primary (44%) and secondary (61%) care facilities.

### Reasons for non-performance of authorized signal functions

**Fig 2:**
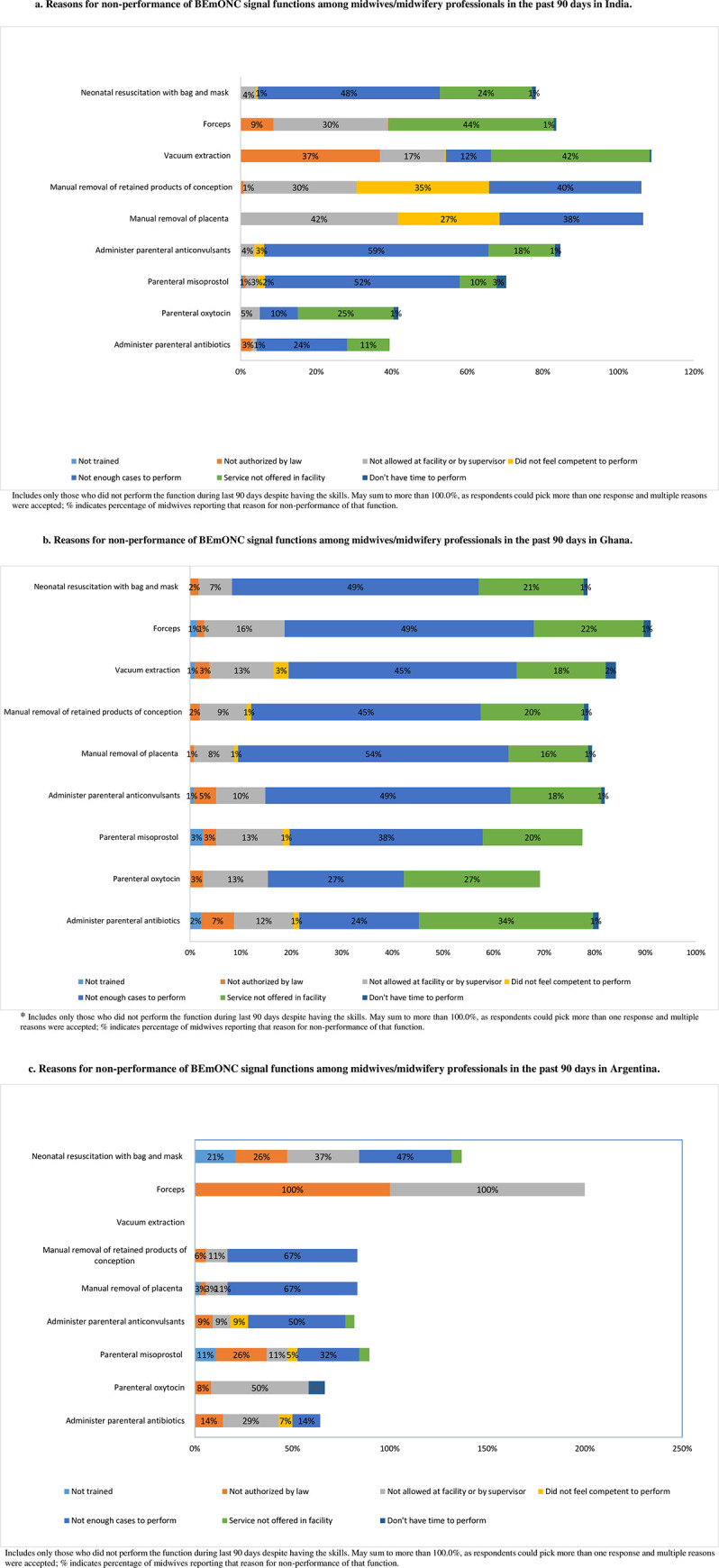
**a.** Reasons for non-performance of BEmONC signal functions among midwives/midwifery professionals in the past 90 days in India. **b.** Reasons for non-performance of BEmONC signal functions among midwives/midwifery professionals in the past 90 days in Ghana. **c.** Reasons for non-performance of BEmONC signal functions among midwives/midwifery professionals in the past 90 days in Argentina. (a-c) Includes only those who did not perform the function during last 90 days despite having the skills. May sum to more than 100.0%, as respondents could pick more than one response and multiple reasons were accepted; % indicates percentage of midwives reporting that reason for non-performance of that function.

When midwives reported non-performance of signal functions for which they had the skills, they were asked the reasons for not performing the function. In India and Ghana, an insufficient number of cases at the facility was the most frequent reason for not performing the authorized function (**Fig 2A and 2B**). For example, nearly 59% of participants with skills in India and 49% in Ghana reported not administering anticonvulsants in the past 90 days due to insufficient cases in their facilities. In Argentina, the major reasons for non-performance were lack of permission by the facility or supervisor as well as an insufficient number of cases (**Fig 2C**).

### Self-reported competency and performance of non-authorized signal functions

Midwifery professionals also reported they had the skills to perform some signal functions for which they were not authorized by the regulatory framework in their country (**Table 5**). For example, midwives in Argentina were not authorized for manual removal of retained products of conception; however, 51% of participants reported that they had the skills to perform the function. Similarly, midwives in Ghana were not authorized to perform forceps delivery, but a quarter of participants reported that they had the skills to do so. Midwives in India were not authorized to perform manual removal of placenta, but 28% of participants reported they had these skills.

Midwives in all three countries also reported performing certain signal functions for which they were not authorized (**Table 5**). For example, in Argentina, 51% of participants who had the skills to manually remove retained products of conception reported that they performed this function in the past 90 days. We observed a similar pattern in Ghana, where 32% of midwives who had the skills for forceps delivery performed the function in the past 90 days. In India, 38% of midwives who had the skills for manual removal of the placenta reported performing the function in the past 90 days.

### Acquisition of skills to perform BEmONC signal functions

When midwives reported having the skills to perform a signal function, they were asked how they acquired the skill. Responses varied by signal function and also by authorization to perform the signal function. In Argentina and Ghana, on the job experience was the most common way midwives reported that they acquired their skills for most signal functions. For example, in Argentina, 81% of midwives with skills to give parenteral oxytocin reported that they acquired this skill on the job, compared to only 64% who reported that they acquired it during pre-service training (S2 Table). Similarly, in Ghana, for all skills except forceps delivery, a higher proportion of midwives reported acquiring the skills through on the job experience than any other modality. Whereas, in India, almost all participants who reported having the skills to perform authorized signal functions reported obtaining their skills through pre-service education. However, on the job experience or in-service training was reported as the means of obtaining the skills for the signal functions that were not authorized in the national regulatory framework.

## Discussion

This study assessed the accuracy of reported data in global monitoring frameworks compared to the national regulatory frameworks for the core global policy indicator, “*Midwives are authorized to deliver basic emergency obstetric and newborn care*” (criterion validity). We also looked for evidence of convergence between midwives’ authorization, skills, and performance (construct validity) to explore the relationship between policy and practice. The results showed variation between national regulatory frameworks and what is reported globally in Countdown and the WHO MNCAH Policy Survey in India, Ghana, and Argentina (although WHO MNCAH Policy Survey data in Ghana matched country source documents).

Survey results from midwives further indicated gaps between midwifery authorization, and skills and performance of BEmONC signal functions. Although BEmONC signal functions are included in the International Confederation of Midwives’ essential competencies for midwifery practice and are listed among the essential interventions that can be carried out by midwives [4, 30], in none of the three study countries were all seven BEmONC signal functions authorized in the national regulatory framework. Our results showed that the midwifery scope of practice varies among countries, with the widest scope of practice in Ghana and the most restricted scope of practice in Argentina. Across all countries, midwifery professionals were only legally allowed to perform one signal function: administer uterotonic drugs. To comply with global guidance or standards, the national scope of work for midwifery professionals should be broadened to authorize all seven signal functions. However, authorization must be accompanied by adequate training for midwives to gain relevant skills and implement them in practice.

Pre-service education emerged as the least reported method for acquisition of skills except in India. For example, in Argentina, 81% of midwives with skills reported that they acquired the skills on the job, whereas only 64% reported that they acquired it during pre-service training. Evidence from other countries revealed similar findings, where midwives reported that they did not gain skills through pre-service education and instead learned skills over time on the job [31, 32]. In many countries, midwifery education is mostly theoretical–students learn through class lectures, with little opportunity to practice those skills during pre-service training [31, 32]. These shortcomings in pre-service training among graduating students could contribute to the limited skills reported among practicing midwives and the lower performance of the signal functions that require those skills. Thus, the lack of acquisition of essential skills during pre-service midwifery training represents an important obstacle in efforts to reduce maternal and perinatal mortality and morbidity globally.

The 2021 State of World’s Midwifery Report documents a shortage of midwives and midwifery professionals globally [33]. The three-gap model (“know-can-do”) suggests a pathway to strengthen the current midwifery workforce [34]. Ensuring that the existing midwifery workforce is educated and trained to global standards and possesses the requisite skills (know), has the authorization to perform essential maternal newborn care interventions (can), and is deployed in settings where there is the clinical need for and the essential supplies to enable actually perform those interventions (do), is a first step toward meeting global coverage projections designed to have an impact on the outcomes of perinatal emergencies.

Midwives in our study reported that they did not recently perform all the signal functions that they are authorized to perform, even when those functions are very basic or expected to be universally applied. For example, routine use of uterotonics to prevent and treat postpartum hemorrhage (PPH) is a staple of evidence-based guidelines [13]. Parenteral oxytocin is the recommended uterotonic agent in clinical guidelines for prevention of PPH through active management of the third stage of labor [35]. All midwives should have the skills to administer parenteral oxytocin. However, our results highlighted that not all midwives have the skill or performed this skill in the recent past. For example, a quarter of the participants in Ghana reported not having the skill and not performing the function in the past 90 days. Misoprostol is one of the 13 essential commodities by the UN Commission on Life-Saving Commodities for Women and Children and is recommended to treat PPH in the absence of oxytocin [35]. Nevertheless, our study found that midwives in Argentina are not authorized to administer misoprostol.

Further, our results suggest that some signal functions may be obsolete based on current practice or clinical evidence. In Argentina and India, midwifery professionals are not authorized to perform assisted vaginal delivery. In Ghana, although midwives are authorized to perform vacuum extraction, less than one-sixth reported performing this function in the recent past. A plausible reason for this could be the declining trend in assisted vaginal delivery due to evidence suggesting an association with increased perinatal morbidity, and thus this function is now often replaced by Cesarean section [36, 37]. Such trends, highlighted in our study results, may warrant reevaluating whether assisted vaginal delivery should be included as a BEmONC signal function going forward.

Our results showed, midwives encounter barriers to performance of some signal functions in their current job despite having both the skills and authorization. For example, only 27% of the midwives in Argentina who participated in the survey reported that they performed manual extraction of the placenta in the past 90 days. While midwives are authorized to perform this skill, the law only permits them to do so when there is no physician present. Most midwives in Argentina work within interdisciplinary teams in their facilities, meaning there may be limited opportunities for them to perform this task despite being skilled and authorized to do so. The fact that specialists perform this task in some settings might impact the ability of primary maternity care providers such as midwives to effectively maintain the essential lifesaving skill. A global WHO survey of midwifery professionals also highlights that hierarchical power dynamics among health professionals can prevent midwives from providing essential services, although they are arguably the most appropriate professional cadre to provide high-quality, primary sexual, reproductive, and maternal newborn care [38, 39]. Midwives who participated in our study reported not being allowed to perform some of the authorized signal functions by the facility administration or by other health professionals or their supervisor in local settings.

Conversely, midwives in our study reported having the skills and performing certain signal functions for which they were not authorized by the national regulatory framework. For example, 38% of participants with skills to manually remove placenta in India reported performing this function in the past 90 days, even though they were not authorized to do so. Plausible reasons for this could be a shortage of health workers or task shifting in these settings, as is the case in rural areas in India, which may have led midwives to obtain those skills and practice them on the job [40]. Thus, midwifery professionals who were not authorized to perform assisted vaginal delivery or manual removal of the placenta or retained products of conception reported having the skills and performing the function in the past 90 days in some of our research settings. This discrepancy between demand for emergency services and regulatory support should be addressed through review and revision of the national scope of work for midwifery professionals.

Self-reported skills and recent performance of signal functions also varied by the level of health system in which midwives worked. This could be because of patient risk stratification and demand for emergency services. For example, in Argentina, a high proportion of the midwives working in secondary care facilities reported performing signal functions; there, high-risk pregnancies are most often referred to higher-level and specialist care. Since midwives are only authorized to perform manual removal of placenta when a physician is not present and in tertiary care facilities there are larger teams and thus higher likelihood of a physician being present, midwives in tertiary facilities may be less able to practice these skills as compared to their colleagues in secondary-level facilities.

Examination of the subsample of midwives who reported performing all authorized BEmONC signal functions highlighted that midwifery professionals had increased opportunity to perform functions in secondary facilities, suggesting that midwives have the ability to practice when opportunity exists. This supports findings by Renfrew and colleagues in the Lancet Midwifery series highlighting that midwifery can support normal reproductive processes and provide first-line treatment of complications [41].

This results of this study should be interpreted in light of certain limitations. First, our study included health workers whose scope of work met the ILO international standard classification of occupations for midwifery professionals regardless of their credentials or job title. There may be some fundamental differences between midwives trained in specialized midwifery programs and the participants in our study, but all participants from all three countries met the study definition based on a widely accepted international classification system. Second, due to the COVID-19 pandemic in the study districts, data were collected through different modalities—through self-administered electronic surveys in Argentina, paper surveys in Ghana, and telephone surveys in India—although the tools were initially designed for face-to-face interviews; this might have introduced some information bias. Further, study participants were midwifery and associate midwifery professionals working on the frontlines in facilities during the global pandemic, where conditions made it more difficult to participate in interviews, potentially affecting the response rate. In India, some of the midwifery and associate midwifery professionals whose names were on the employee rosters could not be reached by telephone in the midst of COVID-19 pandemic, introducing potential selection bias. Third, performance of signal functions and reasons for non-performance were self-reported and thus subject to recall bias. Fourth, the reference period for capturing the practice of signal functions was the past 90 days, chosen to align with the standard timeframe for assessing functionality of EmONC facilities [42]. However, a longer reference period may have yielded different results. Lastly, data collected from four subnational study districts in each research country do not reflect complete national data.

## Conclusion

National regulations were not accurately captured in global monitoring frameworks, posing a threat to criterion validity for this indicator in all three countries. Authorization of midwives to perform all seven BEmONC signal functions is a global indicator; furthermore, it monitors part of a three-part construct in which convergence is assumed—if midwives are authorized to perform signal functions through their national regulatory system, this should trend convergently with their acquisition of the skills to perform those functions, which should correlate with actual performance. We tested that assumption and evaluated validity of the construct that authorization enables midwives and midwifery professionals to effectively deliver emergency care. Our findings indicate construct validity problems in all three participating countries of India, Ghana, and Argentina. First, except for in Ghana, we found that midwives were not authorized to perform all seven BEmONC signal functions. Second, in all three countries midwives did not report having the skills or performing those signal functions for which they were authorized. Finally, midwives reported having skills and actually performing certain signal functions for which they were not authorized. Further, our results suggest gaps in pre-service training for midwives to perform all BEmONC signal functions, which are critical interventions for improving maternal and newborn health and saving lives. The discrepancy between the demand for emergency services that can be provided by midwifery professionals and the lack of authorization by their national regulatory framework as well as training opportunities to acquire basic emergency skills should be addressed through revision of national scopes of work and curricula for midwifery professionals to achieve the objective set through this policy indicator.

## Supporting information

S1 Table Health system level variability in the skills and performance of BEmONC signal functions by midwifery professionals in the last 90 days by country. (DOCX)

S2 Table Midwifery professionals’ acquisition of skills to perform BEmONC signal functions by country. (DOCX)

## Abbreviations

BEmONC: Basic emergency obstetric and neonatal care
EPMM: Ending Preventable Maternal Mortality
ISCO: International Standard Classification of Occupations
ILO: International Labour Organization
LMIS: Low- and Middle-Income Countries
MNCAH: Maternal, newborn, child, and adolescent health
PPH: post-partum hemorrhage
SDG: Sustainable Development Goal
UN: United Nations
WHO: World Health Organization
