# Supplementary material for: Authorization of midwives to perform basic emergency obstetric and newborn care signal functions in Argentina, Ghana, and India: A multi-country validation study of a key global maternal and newborn health indicator
Source: PLoS One. 2023 Apr 20;18(4):e0283029. doi: 10.1371/journal.pone.0283029 (PMC10118111; doi:10.1371/journal.pone.0283029)
Supplement: S1 Table — (DOCX) [file pone.0283029.s001.docx]

**Supplementary Table 1: Health system level variability in the skills and performance of BEmONC Signal Functions by Midwifery professionals in the last 90 days by country**

| **India** | **Facility Type** | | | | | |  |  |
| --- | --- | --- | --- | --- | --- | --- | --- | --- |
|  | **Primary Care** | | **Secondary Care** | | **Tertiary Care** | |  |  |
| **BEmONC Signal Functions** | **Skills % (n)** | **Performance in past 90 days % (n)** | **Skills % (n)** | **Performance in past 90 days % (n)** | **Skills % (n)** | **Performance in past 90 days % (n)** |  |  |
| **Number of Midwives *** | **208** | | **425** | | **133** | |  |  |
| Administer parenteral antibiotic % (n) | 97.6 (203) | 83.74(170) | 94.6 (402) | 90.3(363) | 100.0(133) | 79.0(105) |  |  |
| **Administer uterotonics** |  |  |  |  |  |  |  |  |
| Administer parenteral oxytocin % (n) | 99.6 (207) | 81.2 (168) | 99.5(423) | 88.7 (375) | 99.3 (132) | 81.1 (107) |  |  |
| Administer parenteral misoprostol % (n) | 94.2 (196) | 53.6 (105) | 99.3 (422) | 73.5 (310) | 98.5 (131) | 75.6 (99) |  |  |
| Administer parenteral anticonvulsants % (n) | 90.4 (188) | 36.2 (68) | 93.4 (397) | 44.6 (177) | 100.0 (133) | 42.1 (56) |  |  |
| Manual removal of placenta % (n) | 10.6 (22) | 27.3 (6) | 29.2 (124) | 54.8 (68) | 52.6 (70) | 10.0 (7) |  |  |
| Manual removal of retained products of conception % (n) | 64.9 (135) | 56.3 (76) | 67.3 (286) | 61.9 (177) | 52.6 (70) | 80.0 (56) |  |  |
| **Assisted vaginal delivery** |  |  |  |  |  |  |  |  |
| Vacuum extraction % (n) | 13.9 (29) | 3.5 (1) | 30.8 (131) | 1.5 (2) | 53.4 (21) | 0 (0) |  |  |
| Forceps delivery % (n) | 31.3 (65) | 7.7 (5) | 44.2 (188) | 8.0 (15) | 66.9 (89) | 4.5 (4) |  |  |
| Neonatal resuscitation with bag and mask % (n) | 98.1 (204) | 43.6 (89) | 98.6 (419) | 61.3 (257) | 99.3 (132) | 71.2 (94) |  |  |
| Midwives who report ALL authorized signal functions % (n) | 86.5 (180) | 22.2 (40) | 89.4 (380) | 33.4 (127) | 97.7 (130) | 33.9 (44) |  |  |
| **Argentina** | **Facility Type** | | | | | |  |  |
|  | **Primary Care** | | **Secondary Care** | | **Tertiary Care** | |  |  |
| **BEmONC Signal Functions** | **Skills % (n)** | **Performance in past 90 days % (n)** | **Skills % (n)** | **Performance in past 90 days % (n)** | **Skills % (n)** | **Performance in past 90 days % (n)** |  |  |
| **Number of Midwives *** | **31** | | **27** | | **61** | |  |  |
| Administer parenteral antibiotic % (n) | 61.3 (19) | 79.0 (15) | 77.8 (21) | 95.2 (20) | 70.5 (43) | 67.4 (29) |  |  |
| **Administer uterotonics** |  |  |  |  |  |  |  |  |
| Administer parenteral oxytocin % (n) | 83.9 (26) | 77.0 (20) | 96.3 (26) | 88.5 (23) | 91.8 (56) | 80.4 (45) |  |  |
| Administer parenteral misoprostol % (n) | 48.4 (15) | 46.7 (7) | 70.4 (19) | 68.4 (13) | 63.3 (38) | 52.6 (20) |  |  |
| Administer parenteral anticonvulsants % (n) | 36.7 (11) | 36.4 (4) | 51.8 (14) | 35.7 (5) | 35.6 (21) | 23.8 (5) |  |  |
| Manual removal of placenta % (n) | 54.8 (17) | 17.6 (3) | 55.6 (15) | 33.3 (5) | 68.8 (42) | 26.2 (11) |  |  |
| Manual removal of retained products of conception % (n) | 32.3 (10) | 30.0 (3) | 51.9 (14) | 57.1 (8) | 47.5 (29) | 51.7 (15) |  |  |
| **Assisted vaginal delivery** |  |  |  |  |  |  |  |  |
| Vacuum extraction % (n) | 0.0 (0) | - | 3.7 (1) | 0.00 (0) | 0.0 (0) | - |  |  |
| Forceps delivery % (n) | 0.0 (0) | - | 0.0 (0) | - | 1.6 (1) | 0.00 (0) |  |  |
| Neonatal resuscitation with bag and mask % (n) | 19.4 (6) | 0.00 (0) | 25.9 (7) | 0.00 (0) | 24.6 (15) | 0.00 (0) |  |  |
| ALL signal functions for which they have authorization % (n) | 48.4 (15) | 6.45 (2) | 55.6 (15) | 18.5 (5) | 63.5 (40) | 15.9 (10) |  |  |
| **Ghana** | **Facility Type** | | | | | |  |  |
|  | **Primary Care** | | **Secondary Care** | |  |  |  |  |
| **BEmONC Signal Functions** | **Skills % (n)** | **Performance in past 90 days % (n)** | **Skills % (n)** | **Performance in past 90 days % (n)** |  |  |  |  |
| **Number of Midwives *** | **353** | | **61** | |  |  |  |  |
| Administer parenteral antibiotic % (n) | 71.1 (251) | 66.5 (167) | 95.1 (58) | 77.0 (47) |  |  |  |  |
| **Administer uterotonics** |  |  |  |  |  |  |  |  |
| Administer parenteral oxytocin % (n) | 72.2 (255) | 74.5 (190) | 93.4 (57) | 68.9 (42) |  |  |  |  |
| Administer parenteral misoprostol % (n) | 56.9 (201) | 69.7 (140) | 96.7 (59) | 67.2 (41) |  |  |  |  |
| Administer parenteral anticonvulsants % (n) | 59.8 (211) | 46.9 (99) | 96.7 (59) | 55.7 (34) |  |  |  |  |
| Manual removal of placenta % (n) | 60.1 (212) | 54.2 (115) | 91.8 (56) | 36.1 (22) |  |  |  |  |
| Manual removal of retained products of conception % (n) | 56.9 (201) | 58.2 (117) | 93.4 (57) | 42.6 (26) |  |  |  |  |
| **Assisted vaginal delivery** |  |  |  |  |  |  |  |  |
| Vacuum extraction % (n) | 34.0 (120) | 35.8 (43) | 73.8 (45) | 26.2 (16) |  |  |  |  |
| Forceps delivery % (n) | 22.1 (78) | 32.1 (25) | 42.6 (26) | 13.1 (8) |  |  |  |  |
| Neonatal resuscitation with bag and mask % (n) | 64.3 (227) | 56.8 (129) | 95.1 (58) | 54.1 (33) |  |  |  |  |
| ALL signal functions for which they have authorization % (n) | 15.3 (54) | 20.4 (11) | 26.2 (16) | 8.2 (5) |  |  |  |  |
| *** Total sample, but the percentages are among those who reported to have the skills to perform those tasks** | | | | | |  |  |  |
