# Supplementary material for: Authorization of midwives to perform basic emergency obstetric and newborn care signal functions in Argentina, Ghana, and India: A multi-country validation study of a key global maternal and newborn health indicator
Source: PLoS One. 2023 Apr 20;18(4):e0283029. doi: 10.1371/journal.pone.0283029 (PMC10118111; doi:10.1371/journal.pone.0283029)
Supplement: S2 Table — (DOCX) [file pone.0283029.s002.docx]

**Supplementary Table 2: Midwifery professionals' acquisition of skills to perform BEmONC signal functions by country**

| **BEmONC Signal Functions** | **India** | | | | **Argentina** | | | | **Ghana** | | | |
| --- | --- | --- | --- | --- | --- | --- | --- | --- | --- | --- | --- | --- |
|  | **Pre-Service education** | **In-service** | **On the job experience** | **Refused** | **Pre-Service education** | **In-service** | **On the job experience** | **Refused** | **Pre-Service education** | **In-service** | **On the job experience** | **Refused** |
| Administer parenteral antibiotics % (n) | 100.0(738) | 71.8(530) | 92.7(684) | 0.0(0) | 52.6 (30) | 73.7 (42) | 79.0 (45) | 1.8 (1) | 79.9 (247) | 67.3 (208) | 85.4 (264) | 1.3 (4) |
| **Administer uterotonics** |  |  |  |  |  |  |  |  |  |  |  |  |
| Administer parenteral oxytocin % (n) | 100.0(762) | 69.2(527) | 94.1(717) | 0.0(0) | 63.9 (46) | 80.6 (58) | 80.6 (58) | 1.4 (1) | 81.4 (254) | 66.3 (207) | 87.8 (274) | 1.0 (3) |
| Administer parenteral misoprostol % (n) | 100.0(749) | 69.6(521) | 94.5(708) | 0.0(0) | 36.7 (18) | 73.5 (36) | 85.7 (42) | 2.0 (1) | 79.2 (206) | 64.6 (168) | 87.3 (227) | 1.5 (4) |
| Administer parenteral anticonvulsants % (n) | 100.0(718) | 72.0(517) | 90.1(647) | 0.0(0) | 46.7 (14) | 86.7 (26) | 76.7 (23) | 3.3 (1) | 83 (224) | 66.3 (179) | 83 (224) | 1.9 (5) |
| Manual removal of placenta % (n) | 0.0(0) | 68.1(147) | 95.4(206) | 0.0(0) | 41.2 (21) | 70.6 (36) | 80.9 (41) | 2.0 (1) | 79.5 (213) | 65.7 (176) | 88.1 (236) | 1.5 (4) |
| Manual removal of retained products of conception % (n) | 0.0(0) | 72.7(358) | 92.9(456) | 0.0(0) | 46.2 (18) | 74.4 (29) | 82.1 (32) | 2.6 (1) | 79.1 (204) | 69 (178) | 89.5 (231) | 0.8 (2) |
| **Assisted vaginal delivery** |  |  |  | 0.0(0) |  |  |  |  |  |  |  |  |
| Vacuum extraction % (n) | 0.0(0) | 88.7(205) | 8.7(20) | 0.0(0) | 100.0 (1) | 100.0 (1) | 0.0(0) | 0.0(0) | 76.4 (126) | 66.1 (109) | 80.0 (132) | 0.0 (0) |
| Forceps delivery % (n) | 0.0(0) | 85.4(292) | 9.9(34) | 0.0(0) | 100.0 (1) | 100.0 (1) | 0.0(0) | 0.0(0) | 74.0 (77) | 61.5 (64) | 71.2 (74) | 1.9 (2) |
| Neonatal resuscitation with bag and mask % (n) | 100.0(755) | 68.5(517) | 92.1(695) | 0.0(0) | 52.6 (10) | 80.0 (15) | 73.7 (14) | 0.0(0) | 82.1 (234) | 76.8 (219) | 84.2 (240) | 0.4 (1) |
